# Biomechanical analysis of internal fixation system stability for tibial plateau fractures

**DOI:** 10.3389/fbioe.2023.1199944

**Published:** 2023-06-14

**Authors:** Guoqiang Wei, Xiaofen Niu, Yuan Li, Tingjie Chang, Jianfang Zhang, Haiyan Wang, Xiaohe Li, Yujie He, Ruijiang Wang, Fei Tian, Yangyang Xu

**Affiliations:** ^1^ Department of Rehabilitation Medicine, Changzhi Medical College Affiliated Changzhi People’s Hospital, Changzhi, China; ^2^ Department of Orthopedics, Changzhi Medical College Affiliated Peace Hospital, Changzhi, China; ^3^ Department of Anatomy, School of Basic Medical Sciences, Inner Mongolia Medical University, Hohhot, China; ^4^ Department of Orthopedics, Changzhi Second People’s Hospital, Changzhi, China; ^5^ Department of Health Management, Changzhi Medical College, Changzhi, China; ^6^ Department of Rehabilitation Medicine, Changzhi Medical College Affiliated Peace Hospital, Changzhi, China; ^7^ Beijing Key Laboratory for Design and Evaluation Technology of Advanced Implantable and Interventional Medical Devices, Beijing Advanced Innovation Center for Biomedical Engineering, School of Biological Science and Medical Engineering, Beihang University, Beijing, China

**Keywords:** biomechanical study, finite element analysis, internal fixation, tibial plateau fracture, weight bearing, interfragmentary motion

## Abstract

**Background:** Complex bone plateau fractures have been treated with bilateral plate fixation, but previous research has overemphasized evaluating the effects of internal fixation design, plate position, and screw orientation on fracture fixation stability, neglecting the internal fixation system’s biomechanical properties in postoperative rehabilitation exercises. This study aimed to investigate the mechanical properties of tibial plateau fractures after internal fixation, explore the biomechanical mechanism of the interaction between internal fixation and bone, and make suggestions for early postoperative rehabilitation and postoperative weight-bearing rehabilitation.

**Methods:** By establishing the postoperative tibia model, the standing, walking and running conditions were simulated under three axial loads of 500 N, 1000 N, and 1500 N. Accordingly, finite element analysis (FEA) was performed to analyze the model stiffness, displacement of fractured bone fragments, titanium alloy plate, screw stress distribution, and fatigue properties of the tibia and the internal fixation system under various conditions.

**Results:** The stiffness of the model increased significantly after internal fixation. The anteromedial plate was the most stressed, followed by the posteromedial plate. The screws at the distal end of the lateral plate, the screws at the anteromedial plate platform and the screws at the distal end of the posteromedial plate are under greater stress, but at a safe stress level. The relative displacement of the two medial condylar fracture fragments varied from 0.002–0.072 mm. Fatigue damage does not occur in the internal fixation system. Fatigue injuries develop in the tibia when subjected to cyclic loading, especially when running.

**Conclusion:** The results of this study indicate that the internal fixation system tolerates some of the body’s typical actions and may sustain all or part of the weight early in the postoperative period. In other words, early rehabilitative exercise is recommended, but avoid strenuous exercise such as running.

## 1 Introduction

The optimal treatment of complicated bicondylar tibial plateau fractures has been a source of contention for decades (McNamara et al., 2015), and bicondylar tibial plateau fractures provide several challenges to surgical treatment techniques. To achieve appropriate bone consolidation, surgical implants must offer mechanical stability at the fracture location. As a result, during the healing process, the internal fixation operation should give the best possible placement and fixation of the bone fragment (Bucholz et al., 2010). Bilateral plate fixation is a well-known therapeutic technique with strong biomechanical stability (Lee et al., 2013; Lee et al., 2018). An analysis of complicated Schatzker V/VI fractures revealed that coronal posteromedial split fragments occurred in 28%–74% of cases (Barei et al., 2008; McGonagle et al., 2019), requiring separate posterior fixing (Samsami et al., 2020). In complicated tibial plateau fractures, the incidence and consequent displacement of posteromedial split pieces are commonly underestimated (Hua et al., 2019). The fracture pattern of complicated bicondylar tibial fractures is characterized by a combination of medial coronal and lateral multifragment depression fractures (Eggli et al., 2008). The treatment of tibial plateau fractures needs to be carried out with stable fixation to allow early mobility. The mechanical environment is an essential factor in fracture healing. Surgeons often prescribe a 6- to 8- week non-weight-bearing period following surgery (Burdin, 2013). This time of non-weight-bearing significantly hinders the patient’s function, potentially delaying the outcome and increasing healthcare expenditures. Finally, a lack of mechanical stress in the knee joint may have an impact on the articular cartilage. During short-term reduced loading conditions there was a significant degree of cartilage thinning; in other words, the cartilage underwent some process of atrophy without mechanical stimulation (Eckstein et al., 2006). Previous research has overemphasized the effects of internal fixation design, plate position, and screw orientation on fracture fixation stability (Chen et al., 2017; Djuricic et al., 2022; Ren et al., 2022). However, the biomechanical environment and intensity of postoperative weight-bearing exercise training in complex tibial plateau fractures remain unknown, and more attention should be paid to the biomechanical characteristics of complex tibial plateau fractures postoperatively.

The AO/OTA classification and the Schatzker classification are the most commonly used fracture classifications. Maurice Muller, one of the founders of the Swiss AO/ASIF (Association for the Study of Internal Fixation), established the Comprehensive Classification of Fractures of the Long Bones, which describes the site, morphology, and severity of long bone fractures through a combination of letters and numbers. The American OTA (the Orthopaedic Trauma Association) adopted Professor Muller’s classification system for long bone fractures and applied its classification method and principles to other bones throughout the body to form the AO/OTA fracture classification system. The principles of AO/OTA fracture classification, in general, follow Muller’s principles of long bone fracture classification. By typing, grouping and subgrouping, fractures of one site or segment can have 27 subgroups. Schatzker typing is a proposed six-class classification based on the radiographic plain view of tibial plateau fractures. The strategies for reconstruction of the tibial plateau depend on the fracture lines identified in the preoperative CT scan. The established fracture classifications cannot be used for the definition of operative treatment algorithms for contemporary fracture management. In contrast, these classifications do not consider the 3D manifestation of the fracture offered by CT imaging. Its insufficiency has led to criticism of the idea of treating bicondylar tibial plateau fractures in the context of posteromedial coronal fractures (Pätzold et al., 2017). According to Samsami et al. (2020), the posteromedial split fragment has a significant biomechanical role in the structural rigidity of bicondylar tibial plateau fractures. Therefore, to conceptually comprehend the biomechanical principles of anteromedial fracture block and posteromedial fractured bone fragment fixation, a fracture model is required. Titanium alloy plates and screws serving as the primary biomechanical load-bearing mechanisms are used in the model, which is based on a genuine case with a typical fracture.

This study analyzed the biomechanical characteristics of a bilateral plate fixation method used to treat bicondylar tibial plateau fractures using FEA. The stability of the internal fixation system, the risk of implant failure, and the risk of fracture instability were evaluated under different motion conditions based on the interaction with the mechanical environment. The mechanical behavior of the fractured tibial plateau stability was determined in this way. The model stiffness, implant stress distribution, fracture block stress distribution, displacement distribution, and tibial and screw plate fatigue life were analyzed to assist surgeons and rehabilitation physicians in their evaluation. It is clinically relevant to explore the biomechanical environment features of internal fixation under three conditions common in standing, walking, and running using FEA. Exploring the biomechanical mechanism of the interaction between internal fixation and bone guides the timing of early postoperative rehabilitation. It provides a clinical reference for the stability of the internal fixation system and the safety of rehabilitation sports training after complex bicondylar tibial plateau fractures in the clinic. The use of digital medicine for fracture surgery and rehabilitation training guidance has been well integrated and tried, which is a worthy approach to promote.

## 2 Materials and methods

The research was approved by the Science and Ethics Committee of the School of Biological Science and Medical Engineering at Beihang University (protocol code: BM20220087).

### 2.1 Subject

A Schatzker type V fracture of the tibial plateau was reproduced using computer-aided-design (CAD) based on Computed Tomography (CT) images (female, 28 years old, height 164 cm, body weight 60 kg); the patient has signed an informed consent. This case is a typical fracture presentation and fits well with the research requirements. The surgeon removed the internal fixation from the patient 1.5 years after the surgery.

### 2.2 Surgical technique

The posteromedial incision of the right tibia revealed the fracture end. After the posterior bone block reduction, the Kirschner needle was temporarily fixed, the T-type plate was placed, and four-length screws were drilled in turn. The fractured end is reduced anteriorly at the medial plateau, and a moderate-length tension screw is placed anteriorly to posteriorly. The medial front fracture line is long, the 5-well bone plate is placed, and 4-length screws are drilled in turn. A curved incision was made through the middle and upper lateral segments (see the longitudinal fracture line of the right lateral tibial plateau), and plate was placed and drilled into the length screw fixation.

### 2.3 Creating the base model

**FIGURE 1:**
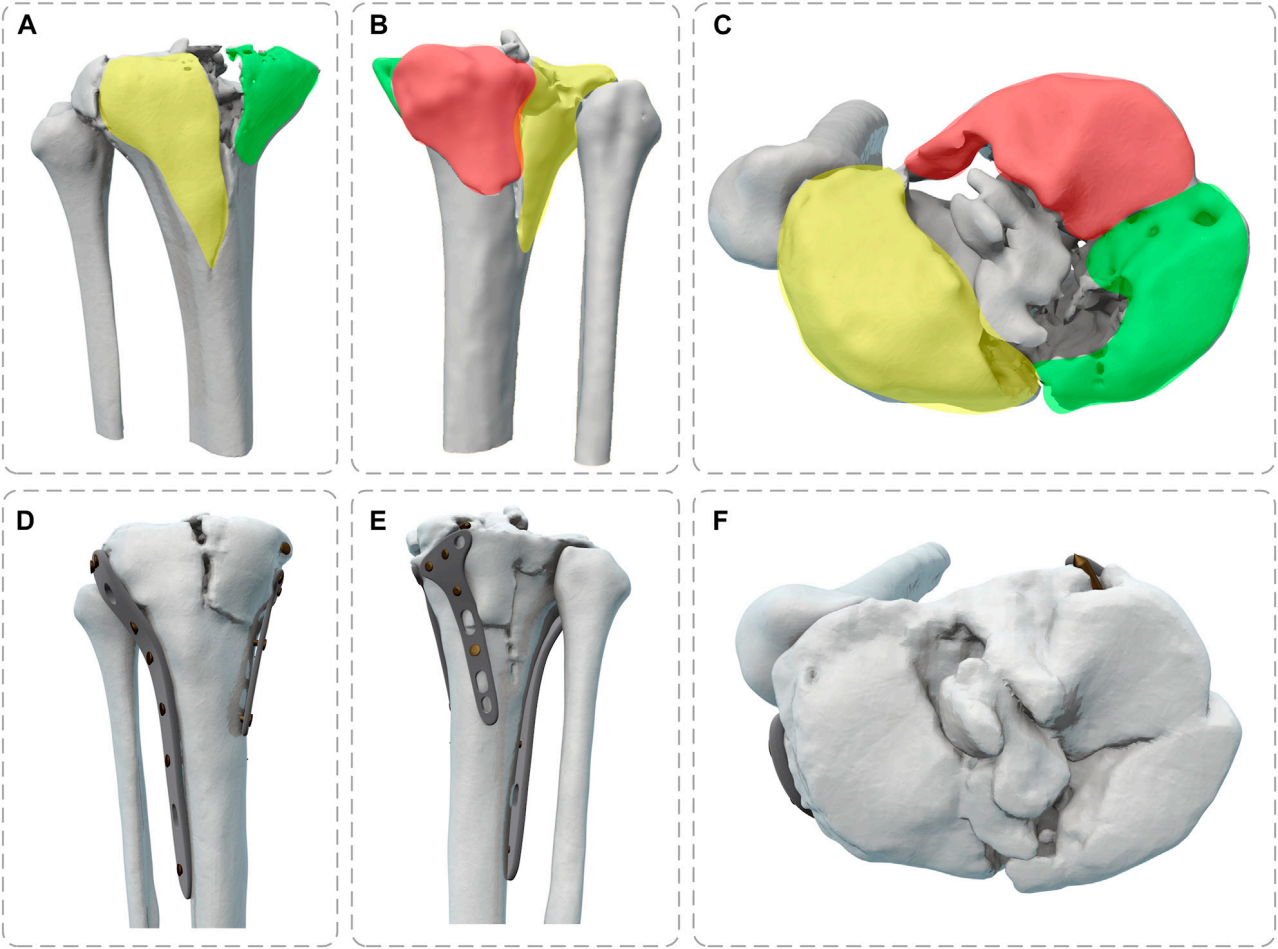
Full view of tibial plateau fracture before and after surgery (Yellow: lateral fracture block, Green: posteromedial fracture block, Red: posteromedial fracture block) **(A,D)** Front View; **(B,E)** Back View; **(C,F)** Top View.

**FIGURE 2:**
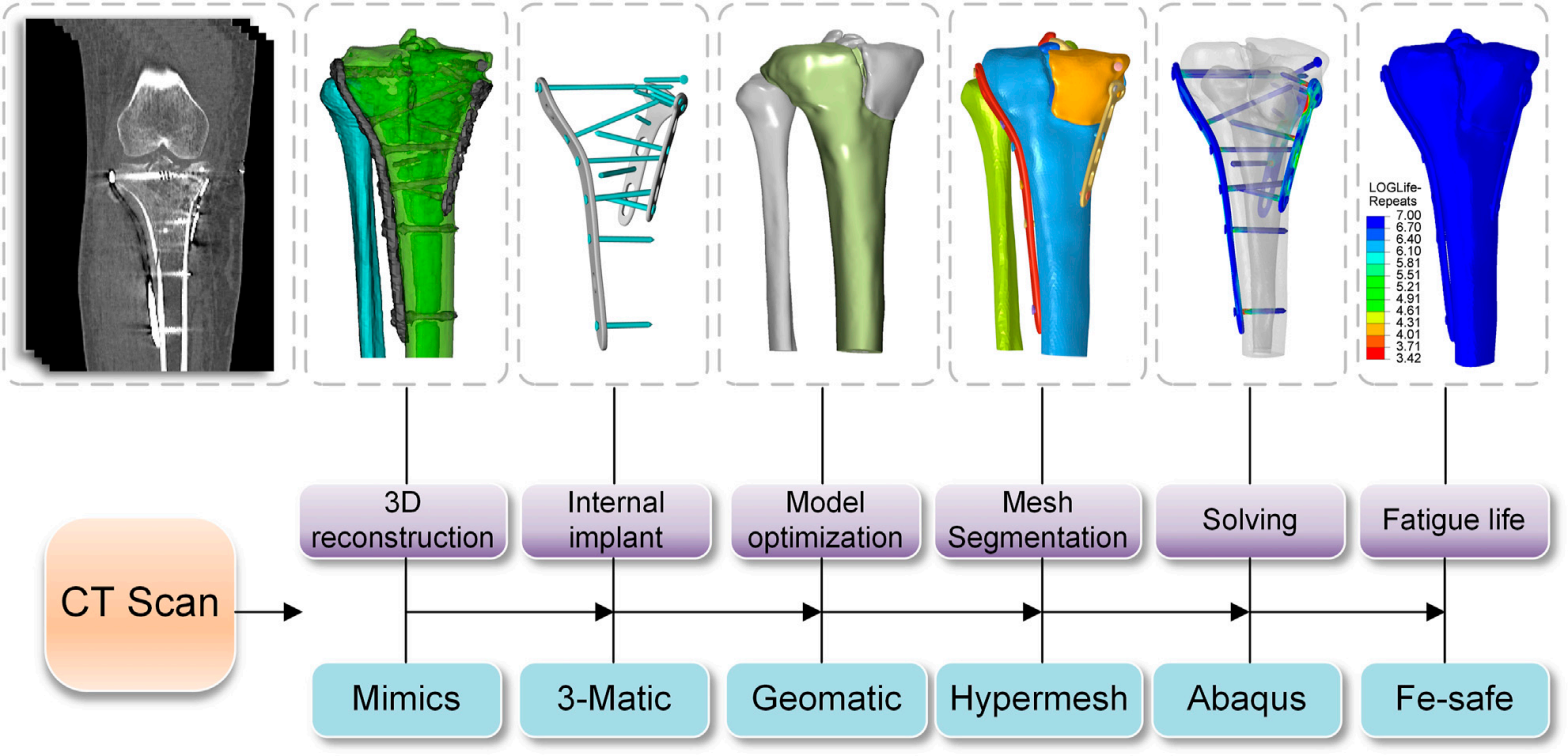
Flow chart of FEA experiments.

Pre- and post-operative high-resolution CT scans of patients to obtain DICOM data (raw HD images from CT scanners) were imported into Mimics 21.0 (Materialise, Leuven, Belgium) to obtain the knee joint structure by threshold segmentation. The Schatzker type V fracture consists of a bicondylar fracture, and the tibial was divided into three fragments (Figure 1). The drawing of screws and plates was done in 3-Matic (Materialise, Leuven, Belgium). To obtain a high-quality tibia-fibula model, the model was processed in Geomagic Studio 2013 (Raindrop Geomagic Inc., Morrisville, NC, United States) for denoising, smoothing, and fitting the surface. The entire model meshed was created in Hypermesh (Altair Engineering Inc., Troy, MI, United States). Define material properties, set boundaries, loading conditions, and computational conditions, and complete the FEA in Abaqus (Simulia, Providence, Rhode Island, United States). Import the calculation file. odb into Fe-safe (Simulia, Providence, Rhode Island, United States) for fatigue life calculation (Figure 2).

**FIGURE 3:**
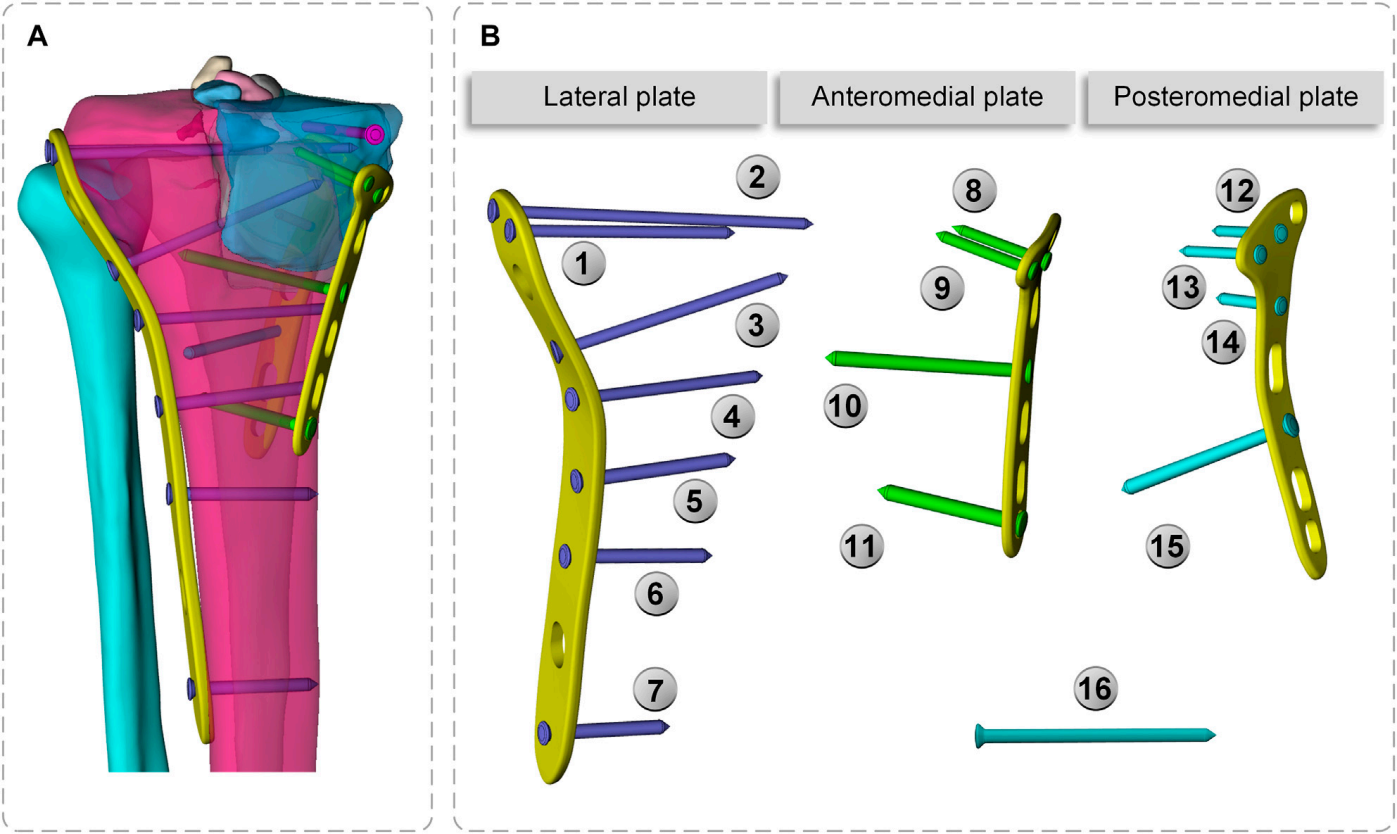
Final CAD models of the bone-implant sets. **(A)** 3D model **(B)** Plates and screws number.

To make the computation more precise, the threaded screws were simplified to smooth screws in this study, the screw holes of the steel plate were obtained using Boolean operations, and contact relations can be easily added (Ren et al., 2022; Zeng et al., 2022). The screws were numbered screws 1–16 (S1–S16), to assist in further analysis (Figure 3). Titanium alloy plate placement: along the longitudinal fracture line of the lateral tibial plateau, an osteotomy plate (lateral plate) was placed along the axial direction of the tibia. Along the anteromedial fracture line, a 5-hole osteotomy plate (posteromedial plate) was placed along the medial tibial condyle obliquely toward the anatomical axis of the tibia, and along the posteromedial fracture line, a T-shaped osteotomy plate (posteromedial plate) was placed along the medial tibial condyle obliquely toward the anatomical axis of the tibia. Screw S16 was a lag screw that was not attached to the plates. In this experiment, we ignored the components that influence knee joint stress, such as ligaments, muscles, and other soft tissues. The stiffness, stress, and displacement distributions of the fractured bone fragments and internal fixation system were determined for various motion states, and a comprehensive report was produced. Select the node with the largest tibial displacement value and plot the force-displacement curve to calculate the model stiffness value (Figure 5A). To achieve the main objective, the final relative displacement was evaluated in eight measurement points located at the fracture edge (Figure 8A). The magnitude of the relative displacement between the two relative nodes of the bone backbone and the fracture fragment was assessed at each site.

### 2.4 Loading conditions and boundary

**FIGURE 4:**
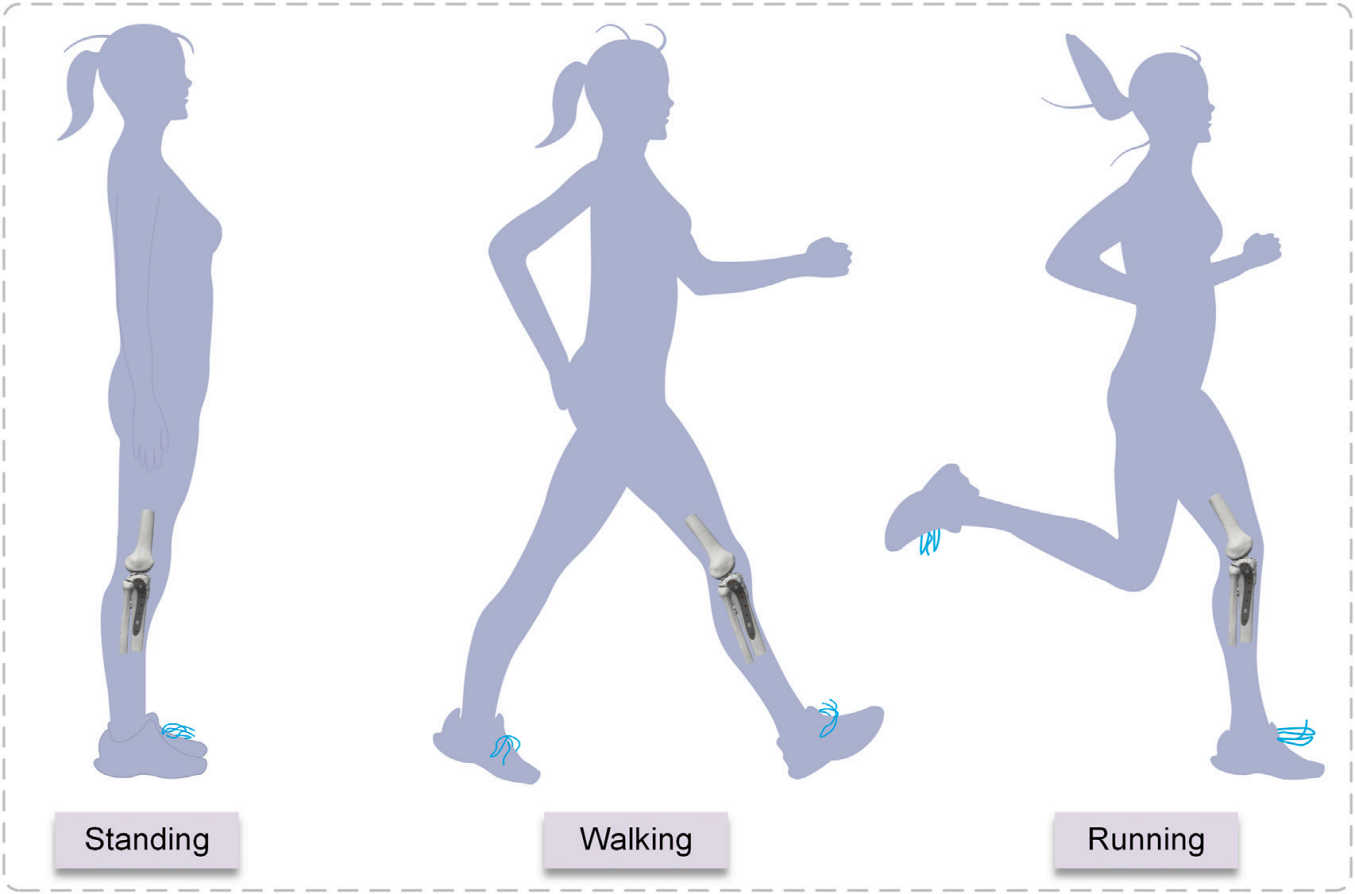
The three conditions of standing, walking, and running.

The biomechanical load on the knee joint during normal gait is approximately two to three times the body weight (Taylor et al., 2004), and the percentage of load on the medial and lateral plateaus is approximately 55% and 45%, respectively (Zhao et al., 2007). When a healthy adult weighing 60 kg is standing, the pressure on the tibial plateau is 60 kg × 9.8 N/kg × 85.6% = 503.33 N (Gao et al., 2022). Walking and running generate two to three times the stress on the tibial plateau than standing (Tai et al., 2009). The majority of individuals spend their days standing, walking, and running. To simulate the stress condition of the tibial plateaus during standing, walking, and running in adults, three axial loads of 500, 1000, and 1500 N were chosen (Figure 4). Due to the incremental nature of the nonlinear analysis, the prescribed loads were applied to the structure in discrete increments. In this experiment, distributed coupling constraints were used to apply loads. Reference points were established medially and laterally on the tibial plateau, and then the reference point was coupled to the reference surface. An axial load was applied at the reference point. The fibula was bonded within the area of the proximal tibial of non-fractured bone by multi-point constraints (Wang et al., 2021). Fixed constraints of the distal tibia and distal fibula. The fractured bone fragments were designed to be frictionless, with a coefficient of friction of 0.3 between the implant and bone and the plate and screws (Koh et al., 2019; Dehoust et al., 2020). When the displacement under load is less than 2 mm, the bone is usually considered undamaged (Haller et al., 2015), and considering the brittle qualities of the material, cortical bone was defined as failing at pressures up to 115 Mpa (Consortium, 2014), and the screws and plates were of titanium alloy with a yield stress of 795 Mpa (Tian et al., 2018; Boluda-Mengod et al., 2021).

### 2.5 Adopted mesh and material properties

The mesh was tetrahedral; the mesh sensitivity analysis was performed under an axial load of 500 N and boundary conditions (see Section 2.4). The final mesh size was obtained by adjusting the mesh size until the displacement value did not fluctuate by more than 1% (Belaid et al., 2018). The resulting mesh is a tetrahedral cell with an average size of 1.5–2 mm, which was consistent with previous research (Dehoust et al., 2020). The node and element numbers were 106,496 and 507,044, respectively. We assigned cortical and cancellous bone separately to the corresponding elastic, homogeneous, and orthotropic properties (Amini et al., 2015). The material properties of the other structures were assumed to have isotropic linear elasticity. Table 1 shows the material properties of all constructs and relevant literature sources.

**TABLE 1:** Material properties.

	Young modulus (MPa)	Poisson’s ration
Cortical bone	E3 = 12,847	υ12 = 0.381
	E2 = 7,098	υ13 = 0.172
	E1 = 6,498	υ23 = 0.160
	G12 = 2,290	υ2 1 = 0.396
	G13 = 2,826	υ31 = 0.376
	G23 = 3,176	υ32 = 3.346
Cancellous bone	E3 = 370.6	υ12 = 0.381
	E2 = 123.4	υ13 = 0.104
	E1 = 123.4	υ23 = 0.104
	G12 = 44.84	υ21 = 0.381
	G13 = 58.18	υ31 = 0.312
	G23 = 58.18	υ32 = 0.312
Titanium alloy plate and screw	E = 110,000	υ = 0.3

### 2.6 Fatigue properties

The fatigue life analysis is based on static analysis, and the results of the static analysis are imported into Fe-safe software. The program defines fatigue damage as several cycles more than 10 to the power of 7 based on the stress amplitude and the estimated number of cycles that fatigue damage may occur. The stress field and strain field are calculated by Abaqus. The axial cyclic loads are set to 500, 1000, and 1500 N, the load amplitudes are set to 0 to +1, the material parameters are set to “E-N Curve,” and the algorithm is selected as the maximum principal stress algorithm.

### 2.7 Validation of the model

The stiffness and Von Mises stress of the model were compared with the experimental data reported in previous studies to validate the plausibility of the model. Gao et al. (2022) showed that the max von Mises stress of the internal fixation system was 69.54, 112.10, and 155.71 MPa for the three conditions of 500, 1000, and 1500 N, respectively. Huang et al. (2015) showed that the maximal stress value in the plate-screw system was 256.20 MPa, while that of the screw was 225.12 MPa. In this study, the max von Mises stress of the plate was 201.52 MPa and the max von Mises stress of the screw was 197.73 MPa. Karunakar et al. (2002) measured stiffness of 2026–2666 N/mm by loading the knee joint after internal fixation. This study produced stiffness values of 2041–2129 N/mm for the model without internal fixation and 2,781–2,868/mm for the model with internal fixation. There were no significant differences in the data, proving that the model was reasonable.

## 3 Results

### 3.1 Stiffness

**FIGURE 5:**
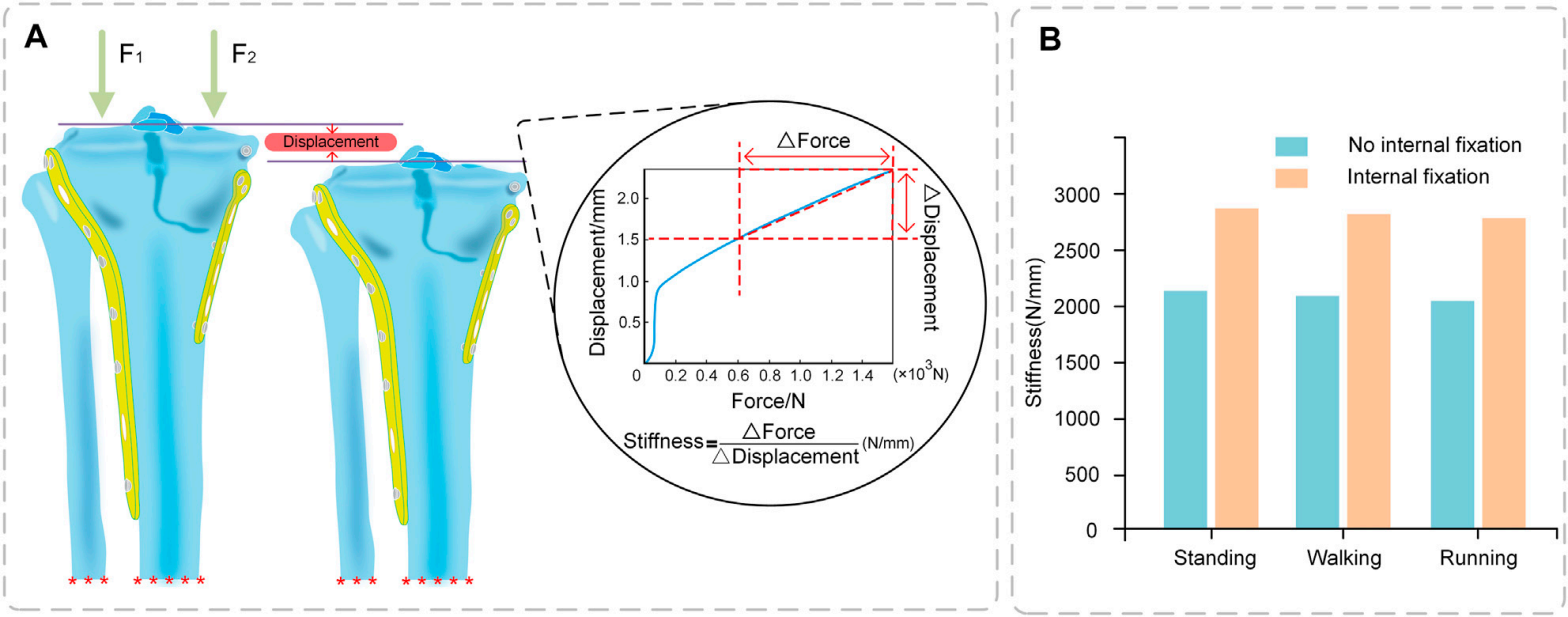
Stiffness of Tibia **(A)** Stiffness calculation (Force-displacement curve); **(B)** Comparison of stiffness between No internal fixation and internal fixation models under different conditions.

The stiffness of the tibia increased significantly after internal fixation by 738.57, 729.96, and 740.67 N/mm in the standing, walking, and running states, respectively. The stiffness values of the tibia did not vary significantly throughout sports (Figure 5B).

### 3.2 Von Mises stress distribution

#### 3.2.1 Von Mises stress of plates

**FIGURE 6:**
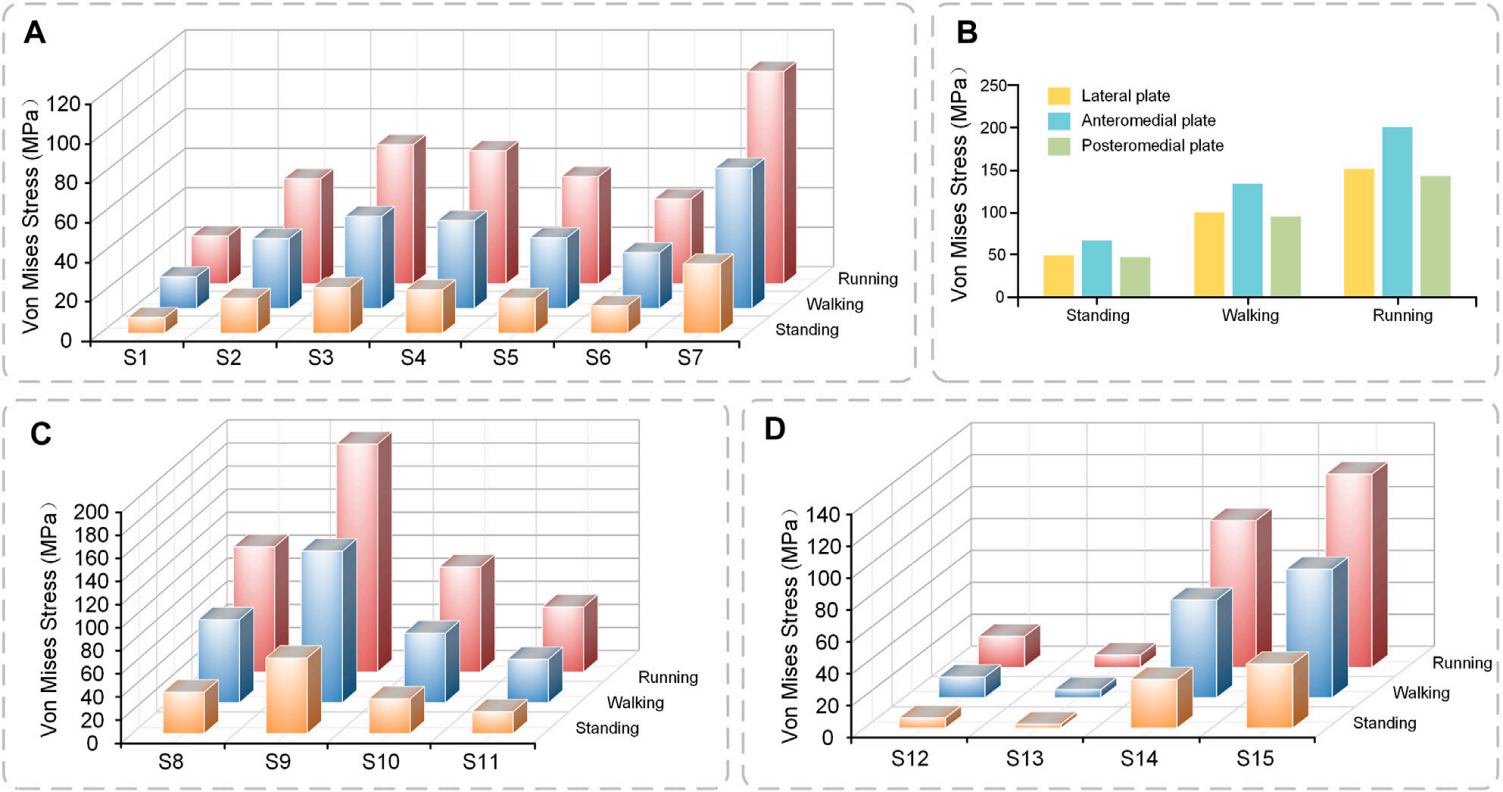
The stress value of plates and screws in postoperative FEMs under different conditions **(A)** Stress value of S1–7 **(B)** Stress value of plates **(C)** Stress value of S8–11 **(D)** Stress value of S12–15.

**FIGURE 7:**
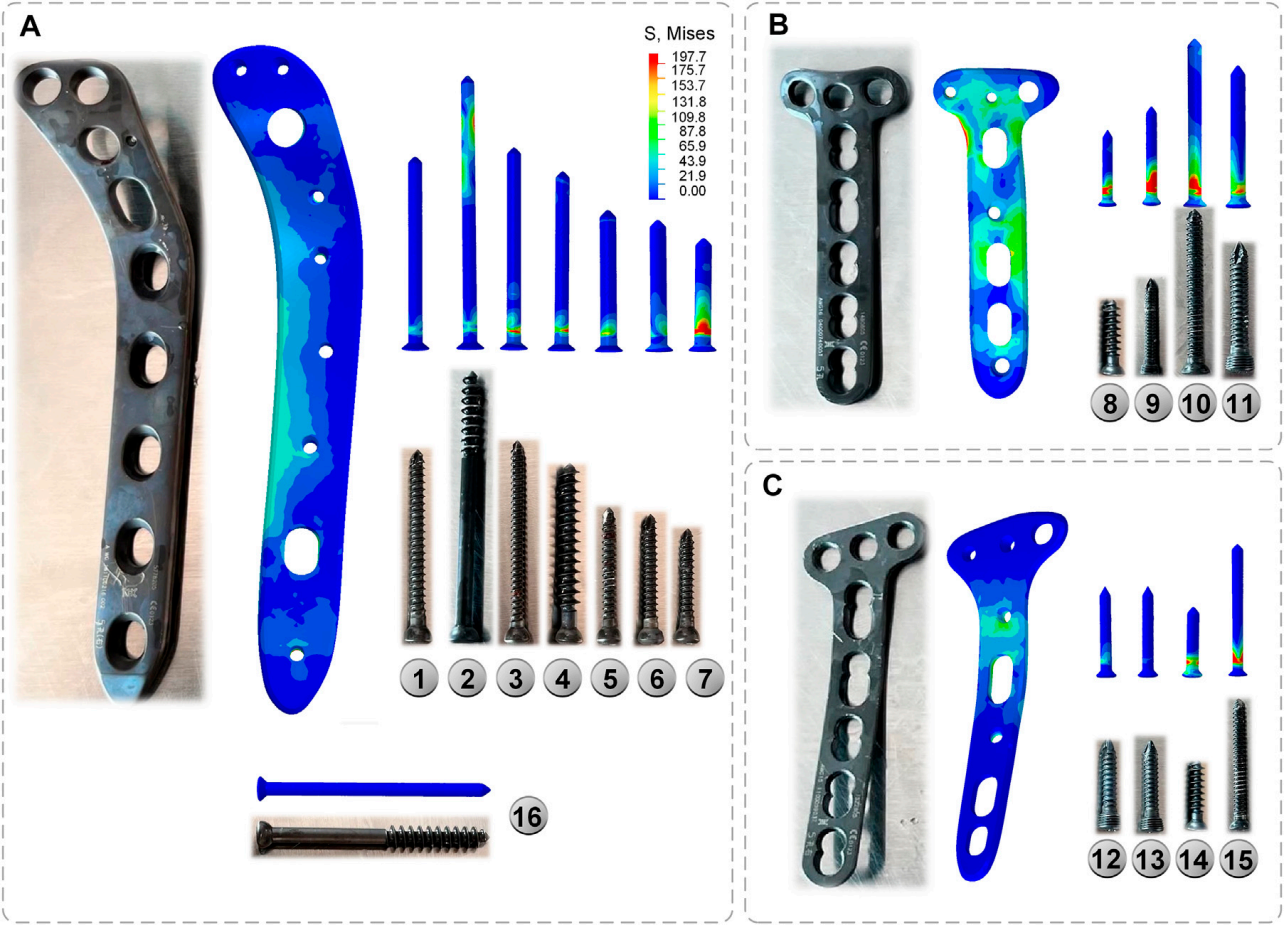
Plates and screws **(A)** Lateral plate and screw 1–7; screw 16 **(B)** Anteromedial plate and screw 8–11 **(C)** Posteromedial plate and screw 12–15.

Under axial loads ranging from 500 to 1500 N, the stresses shared by the plates gradually increase. The anteromedial plate was most stressed, followed by the posteromedial plate, and the lateral plate primarily serves as a support (Figure 6B). The shape of the lateral plate is curved, and the stress cloud diagram shows that the stress of the lateral plate was mostly in the region of the concave edge, with a larger stress at the screw hole at the distal end. The forms of the anteromedial plate and posteromedial plate are T-shaped, and the stress was mostly in the corner of the near end of the plate, PII as a whole exhibits relatively high-stress conditions, but they do not reach the yield stress of titanium alloy and are in the safe stress range (Figure 7).

#### 3.2.2 Von Mises stress of screws

The general trend of each set of screws was comparable in the three conditions of standing, walking, and running, and the screws at the same location rose with the increase in motion intensity. Screw S7 at the most distal end of the lateral plate, has the highest stress, whereas screws S1–S6 exhibit a rising and then declining pattern (Figure 6A). Screws S8 and S9 at the proximal end of the anteromedial plate have the largest stress, whereas the screw at the distal end of the anteromedial plate gradually diminishes (Figure 6C). The stresses in screws S12 and S13 near the proximal end of the posteromedial plate are low, whereas those in screw S14 were rather high (Figure 6D). S16 was not attached to the plates and did not appear in the bar graph. S16 did not experience substantial axial stress, with stress values of 3.08, 6.16, and 9.21 MPa for the three conditions of standing, walking, and running.

Through the stress cloud, similar stress distribution features can be seen, with large stress concentrations near the screw head. For screw S2, stress concentrations were also detected close to the screw tip due to the screw straddling the medial and lateral tibial plateaus (Figure 7).

### 3.3 Displacement distribution

**FIGURE 8:**
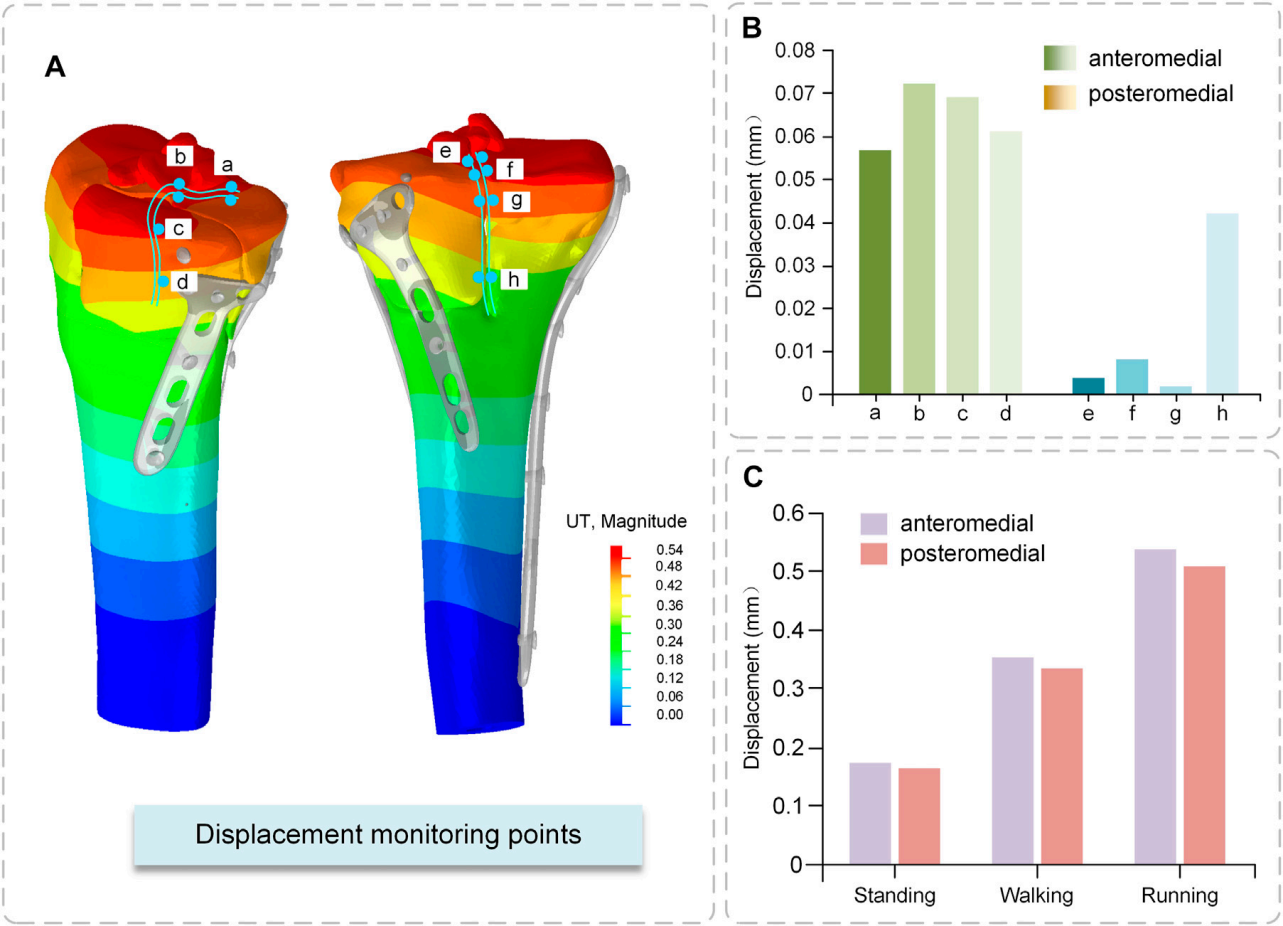
Displacement of fractured bone fragments **(A)** Displacement Cloud Map **(B)** Relative displacement of fractured fragments **(C)** Total displacement of fractured fragments.

The anteromedial bone fragment experienced a large relative displacement of 0.072 mm at the fracture line at the edge of the tibial plateau, extending medially and decreasing in displacement. The posteromedial bone fragment experienced a small relative displacement value and a relatively large displacement value of 0.042 mm at the axial split (Figure 8B).

The total displacement (the maximum value of the cloud map) variation in the anteromedial portion of the fractured bone fragments and the posteromedial portion of the fracture block differs for the two critical fractured bone fragments. The displacement values were somewhat bigger in the anteromedial portion than in the posteromedial portion under different conditions, and the displacement values increased with increasing motion intensity. The largest displacement occurred during running, 0.54 mm for the anteromedial portion and 0.51 mm for the posteromedial portion, neither of which exceeded 2 mm, indicating that the fractured bone fragments were in a stable state (Figure 8C).

### 3.4 Fatigue properties

**FIGURE 9:**
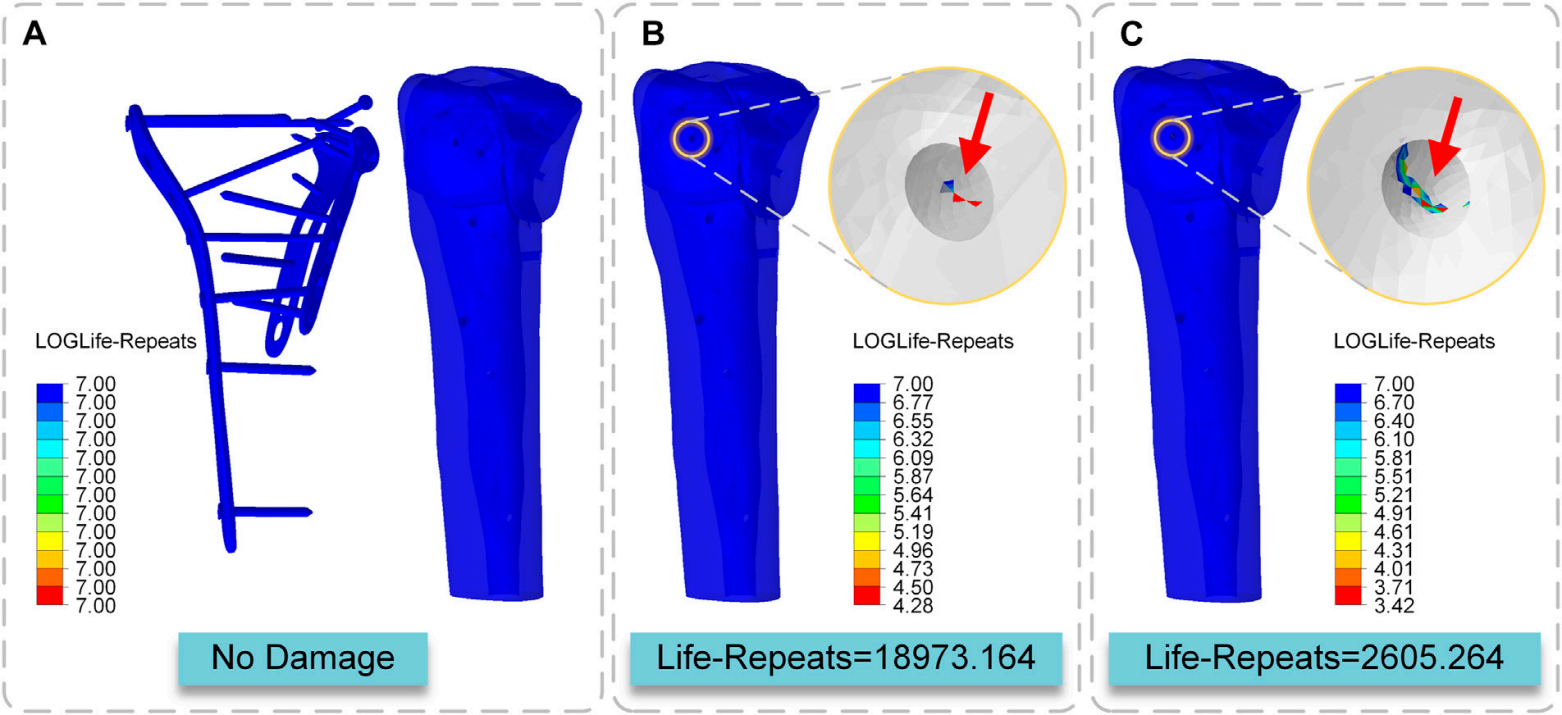
Cloud map of LOGLife-Repeats **(A)** Internal fixation system in three states and the tibia in the standing state **(B)** The tibia in the walking state **(C)** The tibia in the running state.

The fatigue life of the internal fixation system under the action of the cyclic load was more than 10 to the power of 7 times in the three conditions of standing, walking, and running, which may be characterized as no damage. The screw path of S8 was damaged in the walking state, and the minimum LOGlife-Repeats was 4.28, so the fatigue life of this area was 10 to the power of 4.28 = 18973.164 times. In the running state, the screw path of S8 was damaged again, and the minimum LOGlife-Repeats was 3.42, so the fatigue life of this area was 10 to the power of 3.42 = 2605.264 times. The location where fatigue occurs is the location of bone-screw contact (Figure 9).

## 4 Discussion

The surgical treatment of comminuted tibial plateau fractures is challenging. The main purpose of surgery is to provide anatomic restoration of the articular surface and rigid fixation to allow early mobilization (Mueller et al., 2003). The displaced condyle must be reduced for a good clinical outcome, the depressed plateau must be elevated and adequately supported, and early rehabilitation must be encouraged. In clinical practice, surgeons provide conservative recommendations to ensure the solidity of internal fixation by instructing patients to rest in bed. Still, breaking the joint can bring about a loss of joint mobility. This study uses the finite element approach to examine three conditions typical of standing, walking, and running to understand the peculiarities of the internal fixation system’s biomechanical environment. The study provides a theoretical foundation for the viability of early postoperative rehabilitation by examining the biomechanical mechanism of the interaction between internal fixation and bone. This study makes recommendations for rehabilitation, emphasizing that appropriate rehabilitation training is safe and helpful for postoperative rehabilitation whereas intense activity is harmful in the initial postoperative phase. A good combination and attempt of fracture surgery and rehabilitation guidance using a digital medicine approach is a worthy method to be promoted.

In one trial, 32 patients with tibial plateau fractures were treated with internal fixation with plates and either immediate postoperative weight-bearing (*n* = 12) or delayed weight-bearing (*n* = 20), with no fracture displacement in either group and comparable complication rates (Haak et al., 2012). In a large study of 51 patients with comminuted plateau fractures, nearly half were given internal fixation and early partial weight-bearing, with no differences in reoperation rates, post-traumatic osteoarthritis, or pain when compared to those given other external fixation braces and limited weight-bearing. Patients who had early weight-bearing treatment returned to work more frequently than those who had limited weight-bearing (Blauth et al., 2001). Kazemi and Archdeacon (2012), Thewlis et al. (2017) have demonstrated that weight bearing did not have a deleterious impact on the outcome of tibial plateau fractures and may thus be safe for postoperative care. Our study provides a detailed analysis of the internal fixation system’s stability and fatigue characteristics during postoperative rehabilitation exercises, evaluates the mechanical characteristics of tibial plateau fractures after internal fixation, and makes recommendations for postoperative weight-bearing rehabilitation.

This study produced stiffness values of 2041–2129 N/mm for the model without internal fixation and 2,781–2,868 N/mm for the model with internal fixation. Karunakar et al. (2002) reported that the internal fixation model was 2,026–2,666 N/mm, which was similar to the present study. Ion Carrera et al. (2016) reported an internal fixation model of approximately 2,800–3,300 N/mm, which was greater than the present experimental results. Although, the values reported by Ion Carrera et al. (2016) are greater than the results of the present study, the results have the same order of magnitude. One reason may be that the experimental research focused on this comparison is limited. Another reason is that the types of fixation explored in their experiments differed from those used in the current study. It can be seen that the stiffness value significantly increases after implantation of the internal fixation system. Internal fixation implantation greatly improves the stability of the knee joint, which is the basis for the safety of rehabilitative exercise training.

Gao et al. (2022) used three axial loads of 500, 1000, and 1500 N to evaluate the stress of the internal attachment fixation system, and the stress value ranged from 48.92 to 155.71 MPa, similar to the results of this study. Many academics have studied the stability of the internal fixation system, but none have examined the sites of the stress distribution between the plate and the screw. The stress distribution features of the specific steel plate and screw are highlighted in detail in this experiment, and it is noted that the front section of the medial condyle has the greatest stress distribution. The tail of the screw attached to the steel plate has the highest stress, which is consistent with the findings of other investigations (Samsami et al., 2020). On the other hand, the maximum stress of the plate and screw is substantially lower than the maximum yield strength of titanium alloy. This indicates that the strength of the internal fixation system meets the normal activities of the body to some extent.

Displacement monitoring sites A, B, E, and F, which are situated on the top surface of the tibial plateau, are crucial for determining the relative displacement of the fractured bone fragments. This region is related to the distal femoral epiphysis and is covered in ligaments and articular cartilage. As a result, it is critical that this surface recovers optimally to restore joint stability. Independent movement of bone fragments is highly detrimental to fracture fixation. This scenario means that following compressive pressure, each bone fragment travels independently of the others. The posteromedial fractured bone fragments demonstrated a smooth transition of displacement on the fracture boundary, implying increased mechanical stability. Ferre et al. (2022) measured the relative displacement of the distal medial tibial condyle between 0.02 and 0.03 mm. They proposed that locking compression plate fixation of the medial tibial condyle reduced the independent movement of the fracture. In our study, the medial condyle was divided into anterior and posterior parts. The relative motion was 0.002–0.072 mm, which is extremely small and indicates the presence of micromovements between the fracture blocks, but does not affect the weight-bearing function of the bone and is within the safe range.

The implants should be developed to sustain the stress of the broken bone and achieve the needed durability, in addition to mechanical stability and biocompatibility. These implants are subjected to repeated fluctuating loads during regular activities, which can lead to implant fatigue failure. Several studies have looked at implant fatigue failure (Kanchanomai et al., 2008; Schüller et al., 2009; Gervais et al., 2016). These studies suggest that unexpected implant fatigue failure may occur. As a result, while proposing these implants for use in bone-damaged patients, extra consideration should be given to their fatigue characteristics. This study showed no fatigue damage to the implant when standing, walking, or running and that the implant’s strength was enough for regular living activities. Under cyclic stress, however, fatigue damage was found in the tibia. In the standing condition, the bone was entirely safe under the strain. Fatigue damage to the bone occurred after 18973.164 cycles of loading in the walking mode. After 2605.564 cycles of loading in the running state, fatigue damage to the bone was seen. The location where fatigue occurs is the location of bone-screw contact. This experiment did not consider the bone repair factor, which means that in the true case, bone fatigue should appear later than in the experiment, but to some extent, it means that when the stress on the tibia exceeds its body weight numerous times, the likelihood of bone disintegration increases significantly. The choice of rehabilitation training intensity affects bone repair, and the results of this study suggest that excessive loading can cause bone destruction, suggesting that the rehabilitator should choose an appropriate training level. As a result, regular standing weight-bearing and a modest amount of walking weight-bearing are allowed throughout the postoperative rehabilitation period, although the intense activity is discouraged.

Limitations of this study: firstly, bone heterogeneity and biological activity were not considered in the local calculation of bone stresses because bone tissue has a high capacity for repair and adaptation, which may be critical and needs to be investigated. Second, only the effect of axial loading was considered in this study, and shear forces from torsion and bending moment were not considered. Third, the model was simplified by ignoring the effects of the meniscus, cartilage, muscles, and ligaments, as well as by simplifying the threads. All these factors can nonetheless constitute a significant contribution to influencing the biomechanical model, and their contribution has to be studied. This study, combined with the calculation of patient-specific model geometry should be part of the future assessment of immediate bearing capacity after fracture reduction. For this purpose, population-based customization of FEM is both feasible and promising.

## 5 Conclusion

The method of creating a 3D internal fixation system from CT images, the segmentation of the bone model, and the modeling process described in this study demonstrate the high accuracy and usability of 3D virtual objects with mechanical properties. In the case of complicated tibial plateau fractures, the postoperative internal fixation system satisfies some of the body’s normal activities. It can sustain all or part of the weight in a static condition at an early stage. When the load on the tibia exceeds the weight of the body multiple times, the risk of bone deterioration increases significantly. As a result, regular standing weight-bearing and limited amounts of walking weight-bearing are permitted throughout the postoperative rehabilitation phase, but vigorous movements such as running and leaping are discouraged. This study may not be enough to issue complete recommendations for the postoperative period, but it may be a good start to provide some ideas for that process. In the future, we will need more clinical cases to validate this process.

## Data Availability

The original contributions presented in the study are included in the article/supplementary material, further inquiries can be directed to the corresponding authors.
